# Neodiversification of homeologous *CLAVATA1*-like receptor kinase genes in soybean leads to distinct developmental outcomes

**DOI:** 10.1038/s41598-017-08252-y

**Published:** 2017-08-21

**Authors:** Saeid Mirzaei, Jacqueline Batley, Tarik El-Mellouki, Shiming Liu, Khalid Meksem, Brett J. Ferguson, Peter M. Gresshoff

**Affiliations:** 1 Centre for Integrative Legume Research, School of Agriculture and Food Sciences, The University of Queensland, St Lucia, Brisbane, QLD 4072 Australia; 2 School of Biological Sciences, University of Western Australia, Crawley, WA 6009 Australia; 3 Department of Plant, Soil and Agricultural Systems, Southern Illinois University, Carbondale, IL 62901 USA; 4 Department of Biotechnology, Institute of Science, High Technology and Environmental Sciences, Graduate University of Advanced Technology, Kerman, Iran

## Abstract

The CLAVATA pathway that regulates stem cell numbers of the shoot apical meristem has exclusively been studied in Arabidopsis; as such insight into other species is warranted. In this study, a *GmCLV1A* mutant (*F-S562L*) with altered lateral organ development, and two mutants of *GmNARK*, isolated from a Forrest M2 population (EMS-mutated soybean) were studied. *GmCLV1A* and *GmNARK* encode for LRR receptor kinases, and share 92% of protein sequence. While *GmNARK* is critical for systemic regulation of nodulation (new organ made on the root through symbiosis), we show that *GmCLV1A* functions locally and has no apparent function in nodulation or root development. However, a recessive, loss-of-function mutation (*S562L*) in a putative S-glycosylation site of *GmCLV1A* causes stem nodal identity alterations as well as flower and pod abnormalities (deformed flower and pod). The mutant also exhibits a homeotic phenotype, displaying abnormal leaf development/number, vein-derived leaf emergence, and a thick, faciated stem. The mutant phenotype is also temperature-sensitive. Interestingly, a novel truncated version of *GmCLV1A* was identified upstream of *GmCLV1A* that is absent from *GmNARK*, but is present upstream of the *GmNARK* orthologues, *MtSUNN* and *PvNARK*. Taken together, our findings indicate that *GmCLV1A* acts on shoot architecture, whereas *GmNARK*, functions in controlling nodule numbers.

## Introduction

Legumes are an important family of angiosperms as they are able to form a symbiosis with bacteria, named rhizobia that fix atmospheric nitrogen gas^1^. This symbiosis can be highly beneficial as it reduces the nitrogen fertiliser inputs in agriculture. Among the over 18,000 legume species, soybean (*Glycine max* (L) Merr.) is one of the most globally significant crops (2015 annual global yield of 319 million metric tons)^2^. Nearly 75% of its genome is duplicated, or homeologous, due to duplication events occurring ~59 and ~13 million years ago^3^. Following the duplication event, both gene diversification and gene loss have occurred.

The shoot apical meristem (SAM) of plants contains a population of undifferentiated stem cells, which give rise to aerial organs^4–7^. This process is best characterised by the *CLAVATA* (*CLV*) signalling network in Arabidopsis, where receptors CLV1, CLV2, Coryne and RPK2 (Receptor Protein Kinase 2) interact with the peptide ligand CLV3 to regulate the stem cell reservoir^4, 8–10^ through WUSCHEL (a homeodomain transcription factor; WUS) gene^11^. Furthermore, *CLV1* is also involved in regulating stem cell number at the root apical meristem (RAM)^12^ as well as fruit development^13^. *CLV1* and *RPK2* belong to a large family of Leucine-Rich-Repeat (LRR) receptor kinase genes^9, 14^. *CLV2* encodes a LRR receptor protein^15^, Coryne encodes a receptor-like kinase with a short extracellular domain^16^, while *CLV3* encodes a prepropeptide^17^ that is edited and modified into a mature, short (12-13 amino acid) CLE peptide signal (reviewed in Hastwell *et al*.^18^).

Perception of CLV3 restricts the production of the transcription factor WUSCHEL^11, 19^, which promotes stem cell activity and increases the production of CLV3^20, 21^. WUS may function in cytokinin signalling^22^ as the cytokinin response regulator gene *ARR7* is suppressed by WUS. Therefore, a balance in soma and stem cell populations is maintained^23^. These ligand signals, dynamic receptor complexes and phytohormones, shape the plant architecture via short- and long-distance communication.

In Arabidopsis, mutations in *CLV* genes lead to enlarged shoot apices and abnormal floral meristems^24–26^. In soybean, two genes called *GmCLV1A* and *GmNARK* (*Glycine max Nodule Autoregulation Receptor Kinase*; formerly known as *GmCLV1B*), that belong to the LRR receptor kinase protein family, are highly similar to *AtCLV1*. At the amino acid level, GmCLV1A and GmNARK are 92% similar to one another^27, 28^, and 60% similar to AtCLV1.

Mutations in *GmNARK* lead to a super- or hyper-nodulation phenotype due to an inability to inhibit early nodulation events^29–31^. NARK mutant and wild type are similar in nitrogen fixation efficiency, but individual nodules in NARK mutants are smaller than the wild type ones^29^. *GmNARK* orthologues have been identified in *Phaseolus vulgaris, PvNARK* ^32^; *Lotus japonicus, LjHAR1* ^33, 34^; *Medicago truncatula, MtSUNN* ^30^; *Glycine soja, GsNARK* ^27^ and *Pisum sativum*, PsSYM29^33^. These genes act in the shoot and regulate nodule numbers via systemic Autoregulation Of Nodulation (AON) in which early nodulation events prevent further nodule development^1, 35, 36^. They act to perceive root-derived, rhizobia-induced, CLE peptides signals that are highly similar to CLV3^37–39^. In soybean, these signals are called *Rhizobium-Induced CLE* peptides (*RIC1* and *RIC2*)^39^. Interestingly, GmNARK also functions locally in nitrate-regulation of nodulation by perceiving a separate root-derived, nitrate-induced, CLE peptide signal, called *Nitrate-Induced CLE* peptide (NIC1)^36, 39^. In *Lotus japonicus, Too Much Love* (*TML*) (a root factor of nodulation)^40^ and *HAR1* (a shoot factor) constitute the same long-distance signaling that control nodule formation.

The receptors CLV2 in *M. truncatula* ^41^ *, L. japonicus* and *P. sativum* ^42^ and KLAVIER (KLV) in *L. japonicus* ^43^ and Coryne in *M. truncatula* ^41^ may participate in receptor complexes with their respective GmNARK orthologue to perceive the nodulation-suppressing CLE peptides and subsequently trigger the production of a ‘Shoot Derived Inhibitor’ (SDI). SDI then travels to roots where it inhibits early nodule development^44, 45^. The SDI signal may be a shoot-derived cytokinin that acts to regulate cell divisions specifically in the nodule primordia^44, 45^. Recent findings indicate that this may occur through the regulation of miR172c, which down-regulates GmNNC1 transcripts, which in turn negatively regulates the expression of early nodulin *ENOD40* ^46^.

Despite being homeologs and having extremely high amino acid similarity, *GmCLV1A* does not complement *GmNARK* ^27^. In fact, to date the function of GmCLV1A has remained totally unclear. Here, for the first time we report our characterisation of *GmCLV1A*, and provide genetic and developmental analyses of the recently isolated, and unique, soybean mutant having a missense mutation in the *GmCLV1A* gene. The data clearly demonstrate neodiversification of gene function between *GmCLV1A* and its homeologous duplicate, *GmNARK*.

## Results

### Identification and characterisation of *GmNARK* mutants by TILLING

**Figure 1:**
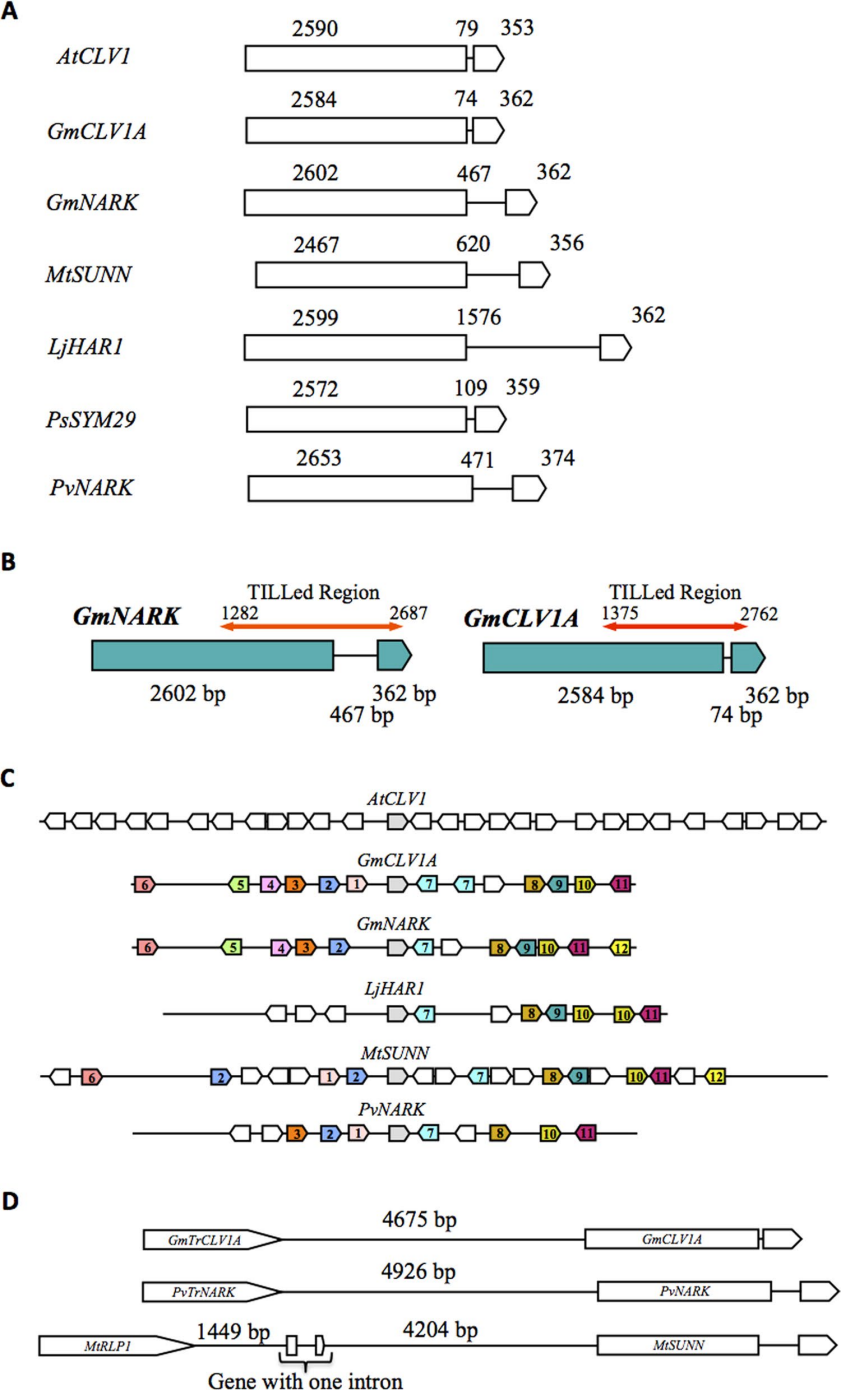
Structure and genomic environments of *CLAVATA1* and AON-related genes. (**A)** Intron and exon positions and sizes of *AtCLV1*, *GmCLV1A, GmNARK, MtSUNN, LjHAR1, PsSYM29* and *PvNARK*. (**B**) TILLed regions of *GmNARK* and *GmCLV1A*. (**C**) Genomic environment of *AtCLV1A*, *GmCLV1A*, *GmNARK*, *LiHAR1*, *MtSUNN* and *PvNARK*; approximately 100 kb is shown. The same number and colour indicates similar genes. The CLV1 and its orthologs in legumes are in grey. The number ‘1’ represents a truncated gene. (**D**) Positioning and size of *GmCLV1A* with *GmTrCLV1A, PvNARK* with *PvTrNARK* and *MtSUNN* with *MtRLP1*.

*GmCLV1A* and GmNARK genes and their protein structure are highly conserved^28^, except for the length and sequence of the intron, which is 74 bp for *GmCLV1A* and 467 bp for *GmNARK* (Fig. 1A). Dot plot analysis failed to reveal any significant similarity between the intron sequences of *GmCLV1A, GmNARK* and *AtCLV1A* (SM, BJF and PMG, unpublished).

TILLING was used to isolate mutants of *GmNARK* from the cultivar Forrest. Two thousand EMS mutagenised Forrest seeds formed a M1 population with 50–70% germination rate. The average mutation frequency in this population was estimated to be one mutation per 150 kb^47^. From this, two pooled cv. Forrest M2 multi-titre plates, consisting of total genomic DNA from 1,536 families that was divided into ‘8 family’ pools, were screened to identify mutations within a 1,406 bp region of the *GmNARK* gene. This region spans parts of the LRR, transmembrane and kinase encoding domains of *GmNARK* and represents about half of the *GmNARK* gene (Fig. 1B). If a DNA pool contained a mutant amplicon, mis-match pairing caused by melting and re-annealing of mixed mutant and wild type DNA, created an ENDO1 cleavage site and two instead of one DNA bands after gene-specific PCR. For a candidate mutant, the apparent mass of the corresponding highlighted bands at the 700 and 800 nm channels were required to equal the total amplicon size. Amplicon-specific primers (Supplementary Table S1) were designed and used for candidate mutant detection and further validation.

In total, twelve *GmNARK* mutants were predicted based on the original TILLING/LiCor output, of which two, F-W677* and *F-H811Q*, were confirmed (Table 1). Mutant allele nomenclature defines the parent cultivar (F for Forrest) and the predicted amino acid alteration (*e.g*., H to Q). A non-sense mutant allele is indicated by an asterisk (*).

**Table 1:** Nodulation and root phenotypes of TILLING mutants in *GmNARK* and *GmCLV1A*.

**Mutant Type**	**Soybean Plant Name**	**Nucleic acid change**	**Amino acid change**	**SIFT score**	**Nodule No. /plant**	**Root Length (cm)**	**Root Weight (g)**	
							**Fresh**	**Dry**
Wild type	Forrest-3	—	—	—	87	33	5.4	0.51
	Forrest-6	—	—	—	95	40	4.2	0.39
	Forrest-9	—	—	—	92	32	5.2	0.51
Known EMS induced hyper-nodulating of Bragg	nts1116-3	GTT > GCT	V837A	0.00	203	28	6.0	0.56
	nts1116-6	GTT > GCT	V837A	0.00	228	35	6.4	0.66
	nts1116-9	GTT > GCT	V837A	0.00	154	32	6.0	0.57
Wild type	Bragg-3	—	—		81	33	6.8	0.60
	Bragg-6	—	—		89	30	9.5	0.80
	Bragg-9	—	—		104	34	7.6	0.61
*GmNARK* TILLING Hyper-nodulating of Forrest	F262-3	TGG > TGA & CTG > GTG	W677* & L829V	0.00 & 0.08	1221	24	2.8	0.28
	F262-6	TGG > TGA & CTG > GTG	W677* & L829V	0.00 & 0.08	1129	27	3.1	0.29
	F262-9	TGG > TGA & CTG > GTG	W677* & L829V	0.00 & 0.08	1269	23	3.2	0.33
*GmNARK* No phenotype	F23_1_A3	CAC > CAT & CAT > CAG	H789 = & H811Q	1.00 & 0.10	50	33	ND	ND
	F23_1_A9	CAC > CAT & CAT > CAG	H789 = & H811Q	1.00 & 0.10	29	29	ND	ND
Clavata1A	NSB1159-3	TCG > TTG	S562L	0.02	68	18	4.3	0.38
	NSB1159-6	TCG > TTG	S562L	0.02	53	35	3.2	0.27
	NSB1159-9	TCG > TTG	S562L	0.02	57	36	3.1	0.27
	NSB1159-12	TCG > TTG	S562L	0.02	9	9	0.2	0.012

Each of the mutants identified contain two closely linked mutations in their *GmNARK* coding sequence; thus, overall there were four mutations identified within the gene. The *F-H811Q* mutant contained a missense mutation (H811Q; located in the kinase domain) and an equal sense mutation (H789 = ), in which the nucleotide, but not the amino acid was changed. Three families were identified which contained these mutations. This mutant did not show any obvious phenotypic differences in root nodule compared to wild type Forrest (Table 1). The severity of the observed phenotypes (data not shown) was consistent with PyMol protein model predictions (data not shown).

**Figure 2:**
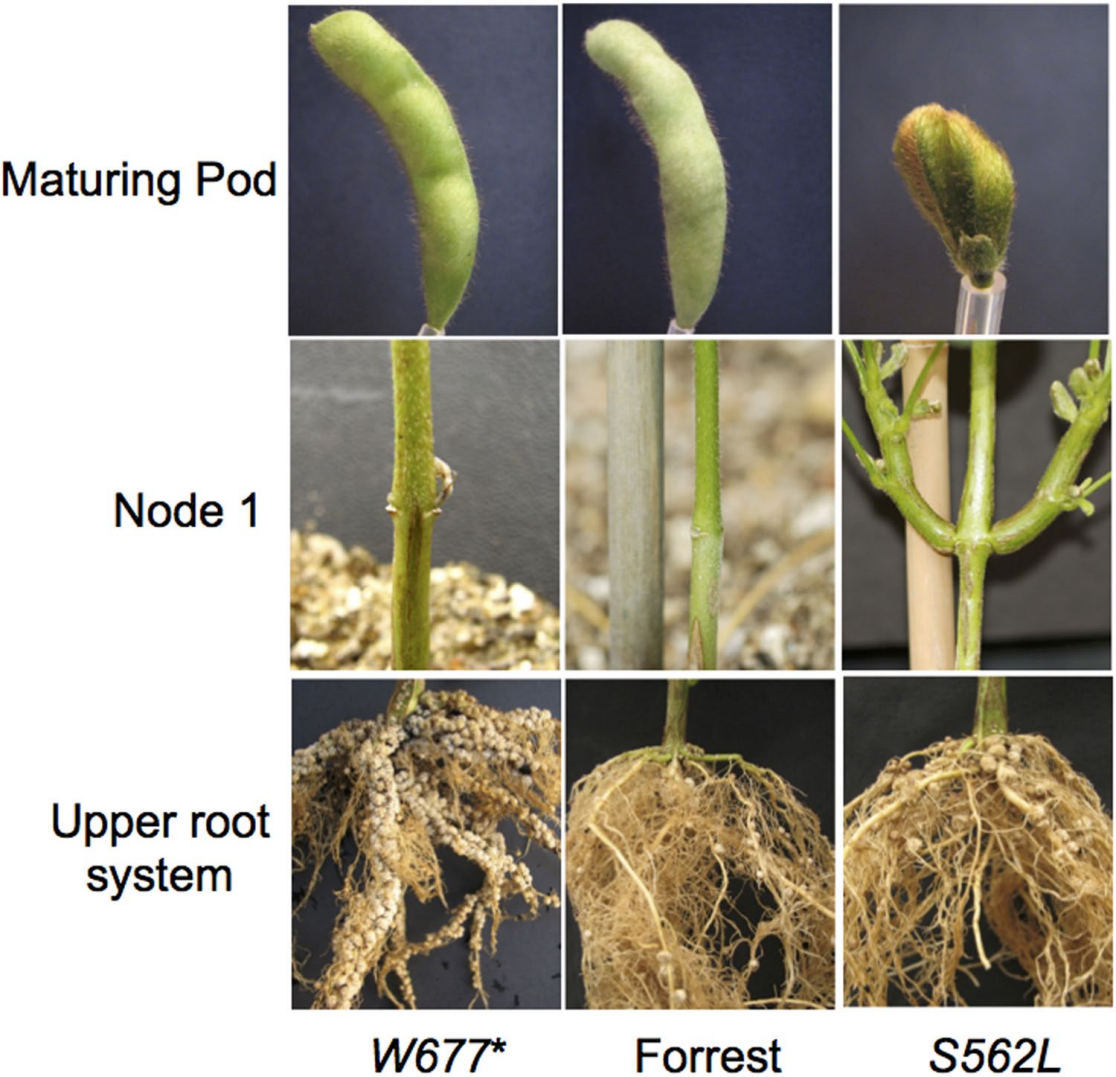
Phenotypes of pod, stem (as demonstrated by cotyledonary node branching) and nodulated root systems of the soybean wild type Forrest, and its TILLING-derived mutants*, Gmclv1a* (*S562L)* and *Gmnark* (W677*).

F-W677* contained a non-sense mutation (W677*), causing a premature stop codon, and also a mis-sense mutation (L829V). The root nodulation phenotype of this mutant showed a dramatic supernodulation phenotype (Fig. 2, left column; here shown at three months after inoculation). The nodule number of the mutant F-W677* was approximately 13 times greater than that of wild type Forrest; the roots were approximately 30% shorter and had a dry weight of 60% of parallel-grown wild type (consistent with previously characterised alleles at the *GmNARK* locus)^48^.

### Identification and characterisation of a *GmCLV1A* mutant by TILLING

**Figure 3:**
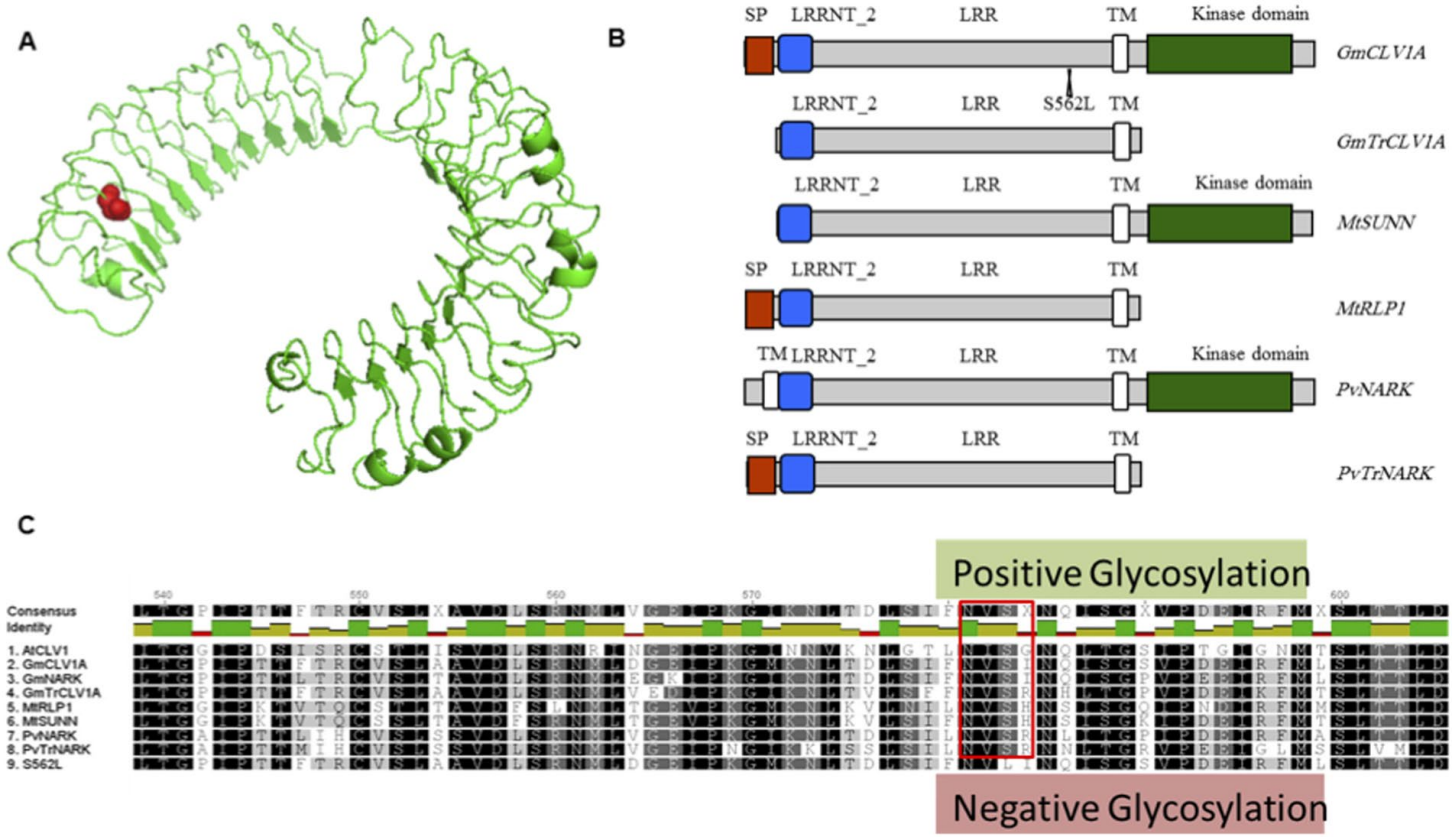
Structural aspects of *GmCLV1A* and GmTrCLV1A. (**A)** Predicted model of the extracellular LRR domain of *GmCLV1A*, including the site of the S562L mis-sense mutation. The amino acid highlighted in red represent the serine of the predicted glycosylation site that is mutated to a leucine in *S562L* (**B)** Predicted protein domains. SP = signal peptide; LRRNT_2 = Leucine-rich repeat N-terminal; TM = Transmembrane domain. (**C)** Protein alignment of the mutated region of S562L compared with that of AtCLV1, *GmCLV1A*, GmTrCLV1A, GmNARK, MtSUNN, and MtRLP1. The red box highlights the predicted glycosylation site.

From the 1,536 M2 families screened, one *GmCLV1A* mutant, *F-S562L*, was confirmed (Fig. 2; right column). This mutant contained a mis-sense mutation (S562L) disrupting a putative serine phosphorylation site in the LRR extracellular receptor domain (Fig. 3A and B). Serine is a polar amino acid and usually contributes to protein phosphorylation, while leucine is a hydrophobic amino acid^49^. The missense mutation interrupts a predicted glycosylation site (Fig. 3C), which would extend hydrophobicity of the protein.

*F-S562L* did not show any obvious differences in its root nodule phenotype compared to wild type Forrest grown under identical conditions (Table 1); however, severe shoot phenotypic differences were observed. The mutant exhibits fewer nodes per plant and increased branching and leaves in the basal nodes (cotyledon, unifoliate and first trifoliate leaf) compared to its wild type, Forrest (Table 2; and Fig. 2; right column). This phenotype was highly related to the fasciated phenotypes of Arabidopsis *CLV1* mutants. No additional mutants of *GmCLV1A* have been found in any mutant population, despite an extensive search, suggesting potential fitness factors and the possibility that some mutations in this gene are lethal, as compared with GmNARK which has many available mutants^32^.

**Table 2:** Nodal abnormalities in the *Gmclv1a* mutant *S562L*. Plants were grown at 28 °C day/night temperature.

**Node**	**Phenotype**	**Forrest** (n = 11)	***Gmclv1a*** (n = 9)	**Gmnark** (n = 8)	Statistical significance
cotyledon	altered leaf number	0^a^	0 ^a^	0 ^a^	*, **
	two branches	2^a^	6^b^	0^a^	
	one branch	4^a^	1^a^	0^a^	
unifoliate	altered leaf number	0^a^	1^a^	0^a^	**
	two branches	9^a^	9^a^	1^b^	
	one branch	0^a^	0^a^	0^a^	
first trifoliate	altered leaf number	2^a^	9^b^	2^a^	**
	two branches	3^a^	8^b^	1^a^	**
	one branch	8^a^	1^b^	6^a^	**
second trifoliate	altered leaf number	0^a^	0^a^	0^a^	*
	two branches	1^a^	0^a^	0^a^	
	one branch	8^a^	4^ab^	2^b^	
third trifoliate	altered leaf number	0^a^	0^a^	0^a^	
	two branches	0^a^	0^a^	0^a^	
	one branch	0^a^	1^a^	0^a^	

Data were collected after 15 weeks and only branches larger than 0.5 cm length were counted. Values represent the number of plants observed with the respective phenotype. Different letters in a row represent statistically significant differences (Duncan test; *P ≤ 0.05; **P ≤ 0.01). Altered leaf number includes a change in leaf morphology and/or number. n: the total number of plants examined.

### *GmCLV1A* genomic environment

To determine whether *GmCLV1A* and *GmNARK* arose from a common ancestor through divergent evolution, or are the result of genome duplications in soybean^3^, the genomic environments within about 50 kb of *GmCLV1A* and *GmNARK* were determined relative to their orthologues, *AtCLV1A*, *LjHAR1* and *MtSUNN*. In all investigated species, the genes reside within highly similar genomic regions (Fig. 1C). *GmCLV1A* and *GmNARK* are located on soybean chromosomes 11 and 12 respectively, proposed to be segmentally duplicated regions of the palaeopolyploid genome^3^ (Fig. 1C).

Of interest, a truncated *GmCLV1A-like* gene was identified 4.7 kb upstream of *GmCLV1A*, and designated here as *GmTruncatedCLV1A* (*GmTrCLV1A*). A similarly truncated gene called *MtRLP1* ^30^ is located 6.2 kb upstream of *MtSUNN* in *Medicago truncatula*, and *PvTrNARK* is a truncated gene located 4.9 kb upstream of *PvNARK* in common bean (Fig. 1D)^32^. All of these truncated versions of the genes has high nucleotide sequence identity to the receptor and transmembrane portions of their genes and lose the kinase domain. It seems that they lead to similar proteins in different legumes are involved in similar biological pathways. However, investigations into *GmNARK* and *LjHAR1* indicated that these genes do not have a truncated copy located upstream, suggesting evolutionary deletion, or independent origins of the truncation. Either way, the function of the truncated copies, if any, remains unknown.

### *GmCLV1A* and *GmTrCLV1A* expression

**Figure 4:**
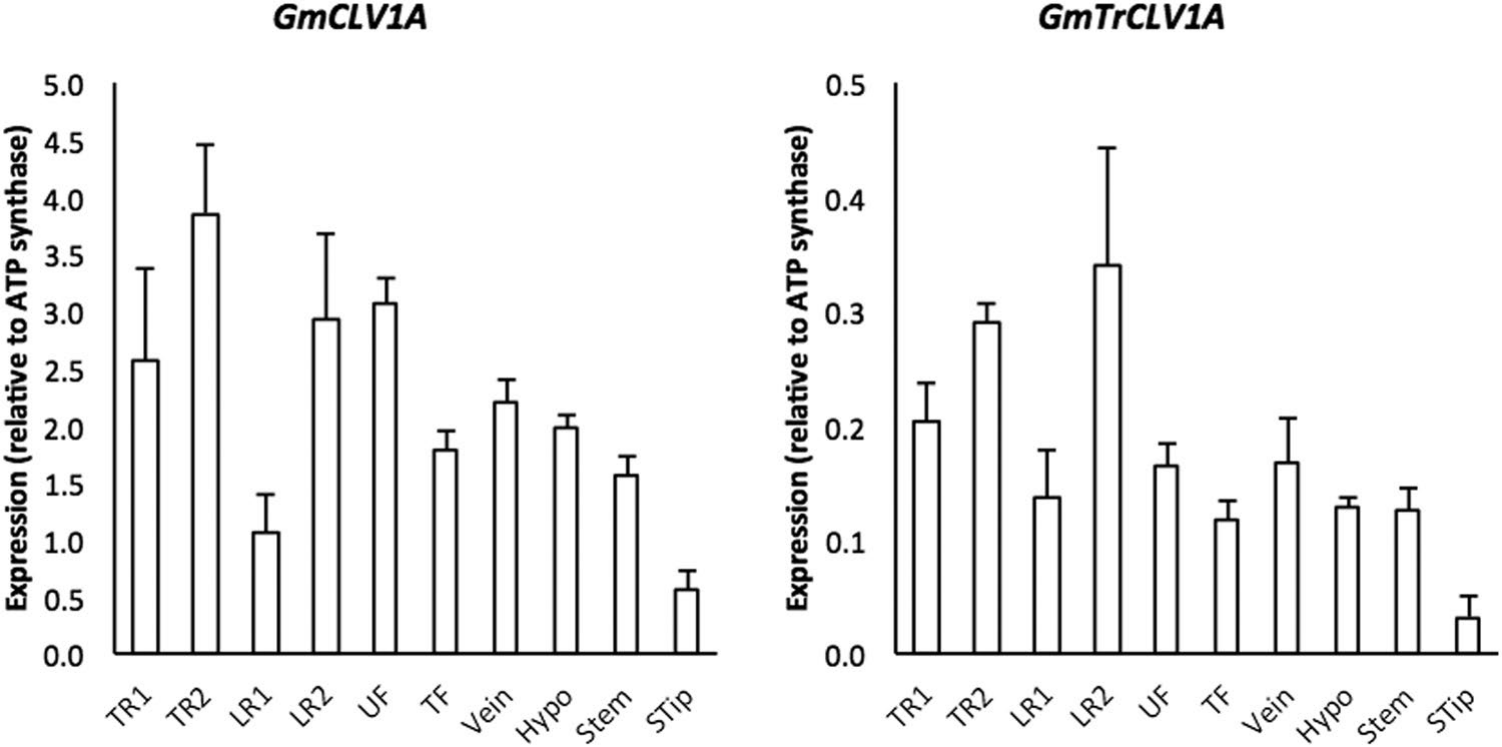
Transcript levels of *GmCLV1A* and *GmTrCLV1A* in various tissues of 14 day-old, uninoculated soybean plants. Values were measured using qRT-PCR; n = 3 biological replicates per tissue; error bars indicate SE. TR1 = first 2 cm from taproot tip; TR2 = second 2 cm from taproot tip; LR1 = first 2 cm from lateral root tip; LR2 = second 2 cm from lateral root tip; UF = unifoliate leaf; TF = trifoliate leaf; Vein = vein of trifoliate leaf; Hypo = hypocotyls; Stem = stem above hypocotyl; STip = shoot tip. Note the 10-fold difference in scale.

Steady-state mRNA levels of *GmCLV1A* and *GmTrCLV1A* were investigated in root, leaf and shoot tissues of un-inoculated soybean plants. *GmCLV1A* and *GmTrCLV1A* were expressed in all tissues analysed, including the shoot tip (Fig. 4). *GmTrCLV1A* was expressed at a much lower level compared with *GmCLV1A* (Fig. 4), which is consistent with *MtRLP1* expression^30^. Moreover, *GmTrCLV1A* expression was consistent with transcriptome data available in the soybean gene atlas (http://soybase.org/soyseq) and soybean eFP browser (http://www.bar.utoronto.ca/efpsoybean/cgi-bin/efpWeb.cgi). *GmNARK*, was also expressed throughout the plant, but not at a high level in the shoot tip (consistent with Nontachalyapoom, S. *et al*.^50^).

### *GmCLV1A* controls shoot nodal identity patterns

**Figure 5:**
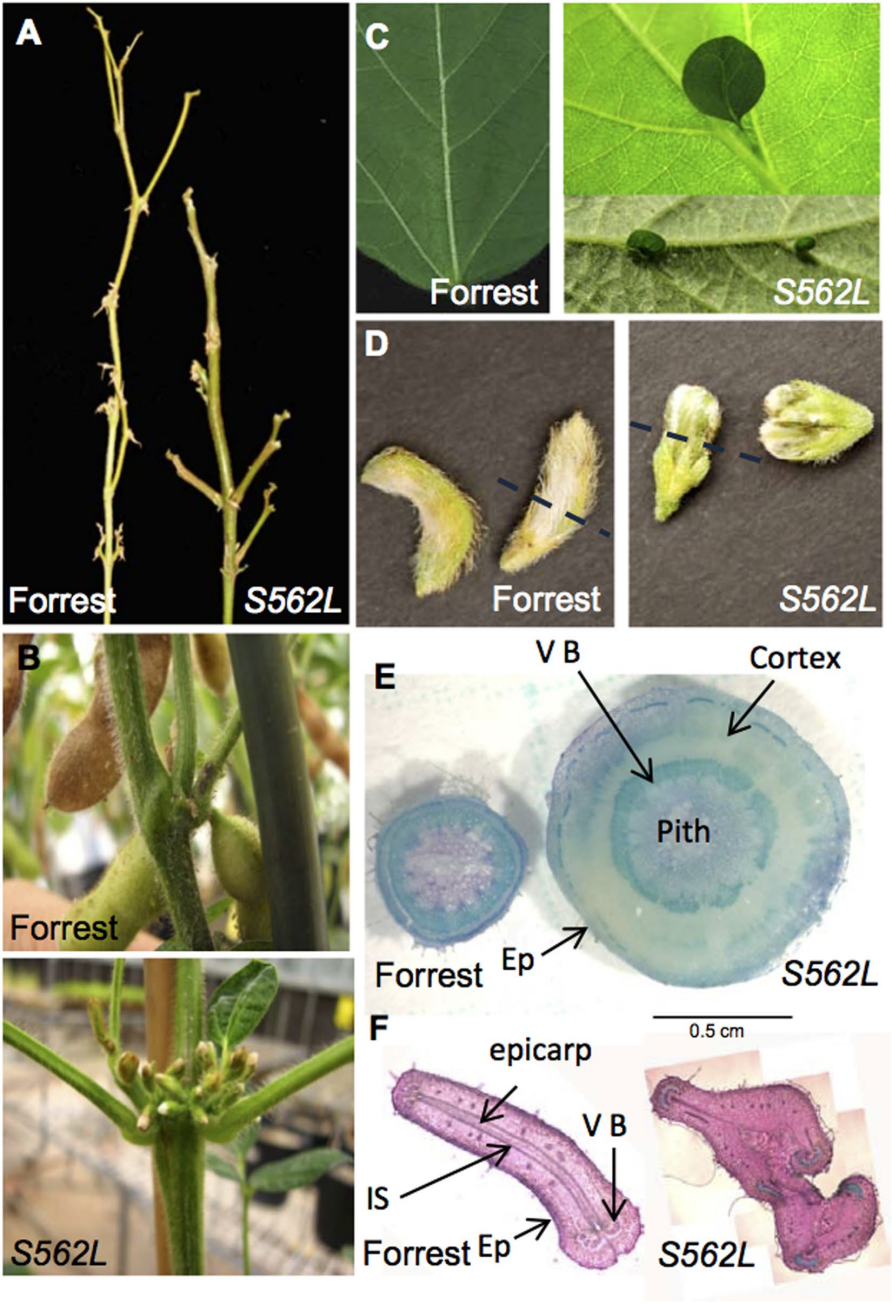
Macro- and microscopic phenotypes of the soybean wild type Forrest, and its mutant *S562L*. (**A)** Stem thickness of 5 month-old plants (plants were intentionally defoliated to enhance visibility of stem architecture); (**B)** First trifoliate node showing fasciation and excessive flowering in the mutant; (**C)** Vein-bladed leaf structures on the underside of *Gmclv1a* mutant leaves. (**D)** Young pod morphology (dashed line indicates the position of the cross-section seen in (**F**). (**E)** Stem section at node 4 of Forrest and *S562L* mutant plants (4 month-old). (**F)** Young pod cross-sections. Note the bifurcated, deformed pod of the *S562L* mutant. VB = Vascular bundle; Ep = Epidermis; IS = Inner sclerenchyma.

The *S562L* mutant exhibits an environmentally influenced phenotype with abnormal leaf development and number (Fig. 5), similar to the homeotic mutant of pea, *Pscochleata* ^51, 52^. Unlike *Pscochleata*, *S562L* also displays a fasciated phenotype, with thick, woody stems, and bifurcated pods that often abort, especially at basal nodes (Fig. 5A,B and D–F). In addition, *S562L* exhibits significantly increased lateral shoot branching (Figs 2 and 5B, Table 2). This is consistent with the differential expression of many genes in the apical meristem of the mutant compared with its wild-type parent, which may cause altered phenotypes^53^. Strikingly, the underside of some leaves of more mature (8-12 week old) mutant *S562L* plants produced small leaf-like structures emanating from their veins (vein-bladed; Fig. 5C). These unique leaf-like structures emerge after flowering and are exclusively located at the junction of the main and lateral veins, sometimes occurring in clusters.

### Segregation of *GmCLV1A* in a *S562*L x Forrest population

**Figure 6:**
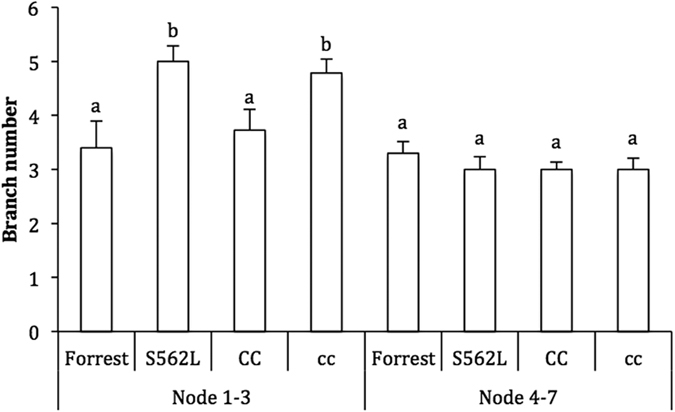
Branching phenotype of 4 week-old soybean cv. Forrest, its mutant *Gmclv1a* (*S562L*), and F_2_ segregants from a cross between them. CC = wild-type segregants; cc = *Gmclv1a* segregants. Forrest n = 10, *S562L* n = 9, CC n = 11 and cc n = 14. Error bars indicate SE. Different letters above bars represent statistically significant differences (Student’s *t* test; *P* ≤ 0.05).

Crossing *S562L* × Forrest (wild type), and segregation was determined in the F_2_ generation. The *GmCLV1A* phenotype was complemented in all F_1_ plants. Fifty F_2_ plants were subsequently derived from self-pollination of a single F_1_ plant. These exhibited the predicted Mendelian ratio for a single gene: 1:2:1 (Fig. 6; χ^2^ = 0.39, *p* > 0.05). In the F_2_ generation, *Gmclv1a* homozygous mutants produced significantly more branches at the first 3 nodes (juvenile phase) compared to wild-type Forrest and wild-type homozygous segregants. Branching at later nodes was similar among all lines (Fig. 6). This confirms that the phenotype segregates with the genotype, demonstrating that the *Gmclv1a* mutation could be the cause of the observed developmental changes.

### Local and systemic function of *GmCLV1A*

**Figure 7:**
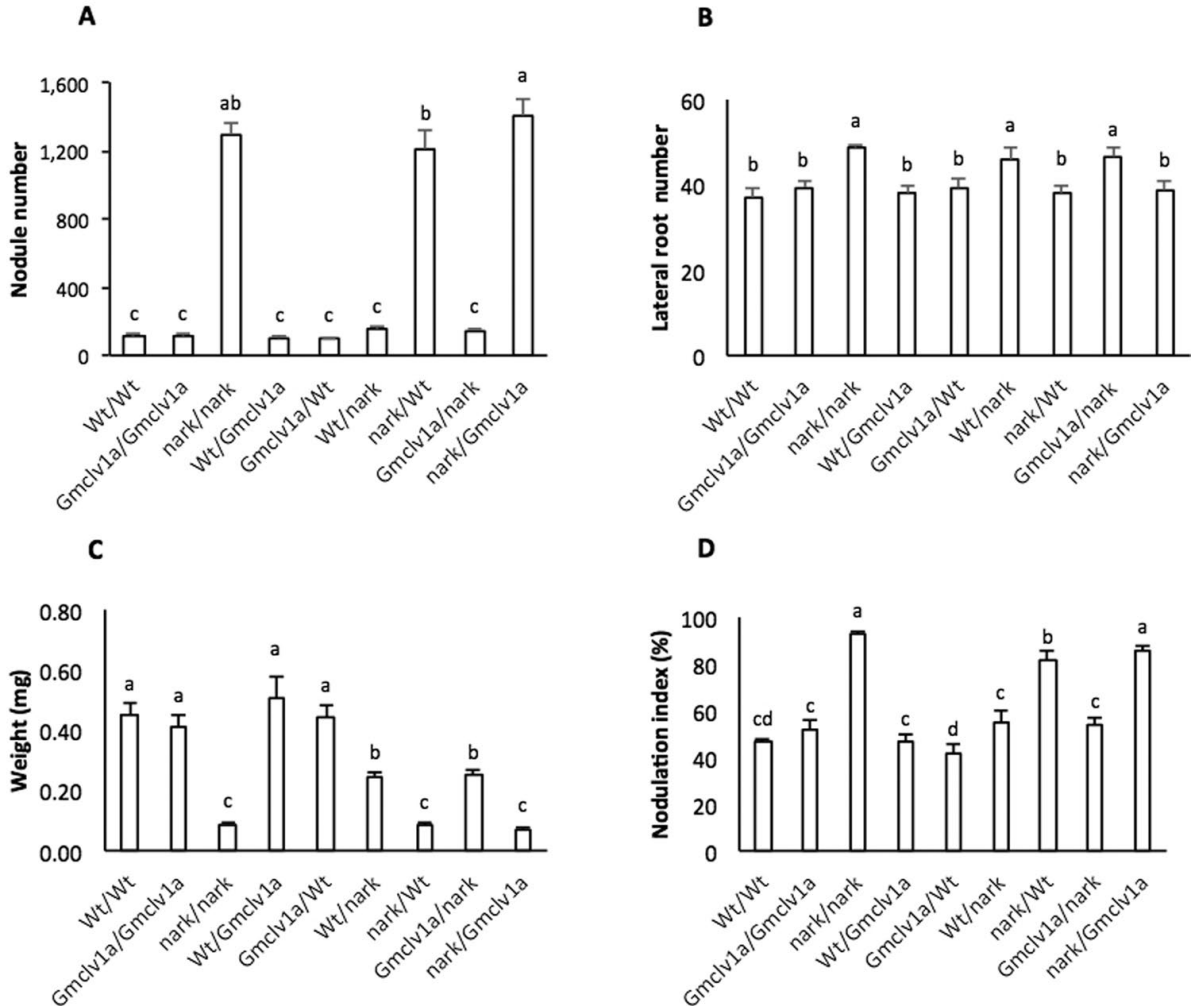
Phenotypes of reciprocally grafted (scion/rootstock) plants between wild-type soybean cv. Forrest and its mutants *Gmclv1a* (*S562L*) *Gmnark* (W677*). Plants were grafted 12 days after sowing. Data were collected 45 days later. (**A)** Nodule number per plant; (**B)** lateral root number per plant (in the 5–15 cm region below the crown); (**C)** average nodule weight; and (**D)** nodulation index (*i.e*., % of root nodulated). Different letters above the bar represent statistically significant differences (Duncan test; *P* ≤ 0.05). Error bars indicate SE.

Hetero-grafting the *S562L* mutant and parent Forrest plants, or the Gmnark W677* supernodulating mutant, showed that *S562L* has no root- or shoot-effect on nodule number, lateral root number, nodule size, and nodulation index (nodulated portion of the root; Fig. 7). In contrast, W677* strongly controls the nodule number (Fig. 7A) and nodulation index through the shoot, consistent with previous reports for *GmNARK* mutants^35^. Moreover, W677* had a pronounced root-controlled effect on lateral root formation, with an additive effect when shoots were W677*, which is also consistent with other *GmNARK* mutants^54^ (Fig. 7B). All combinations having W677* in either the root or shoot exhibited smaller nodules; however, this effect was pleiotropically stronger when W677* was in the shoot (Fig. 7C).

### *Gmclv1a* induced phenotypes are temperature regulated

**Figure 8:**
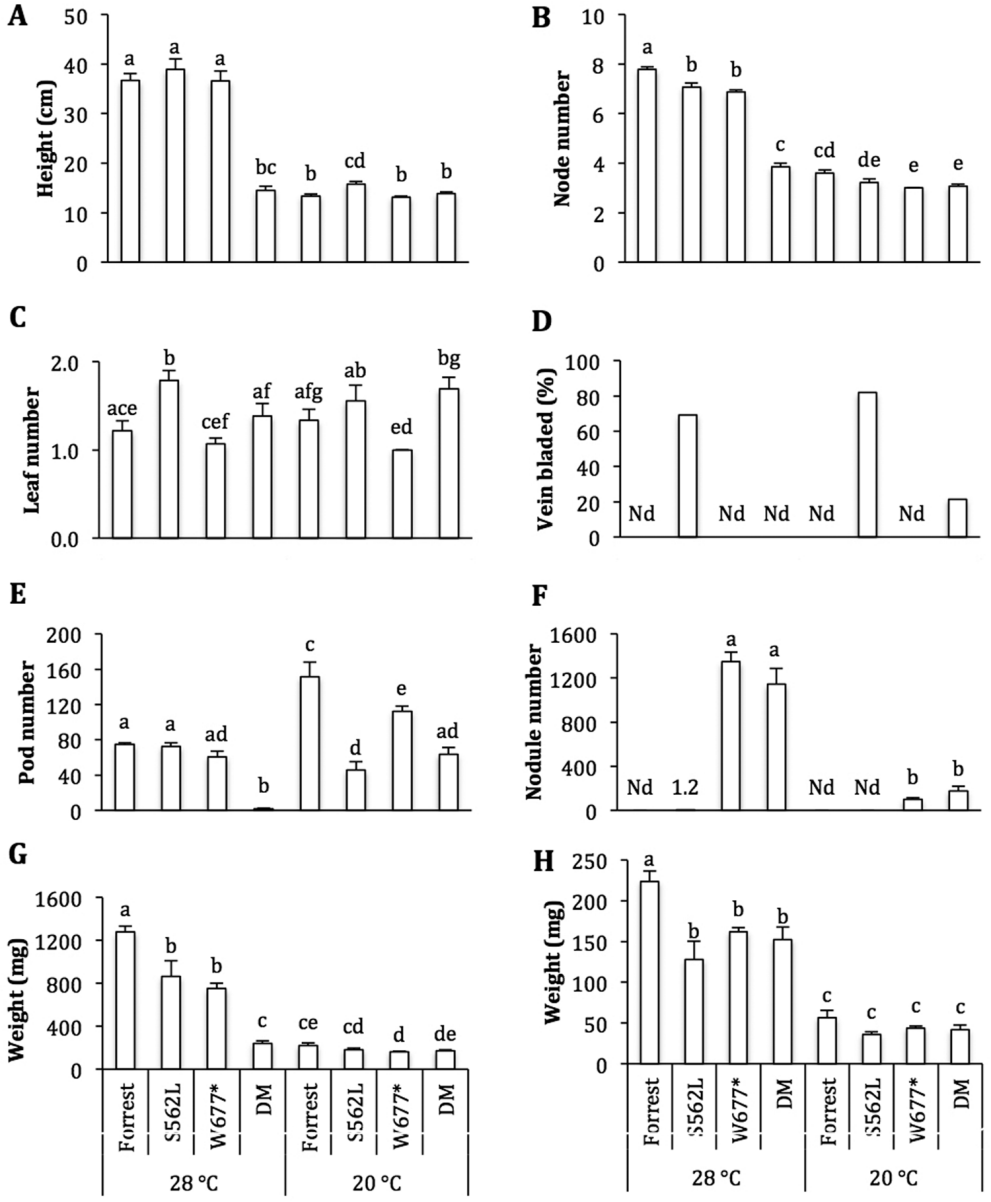
Temperature influence on phenotypes of wild-type soybean cv. Forrest, and its mutants *Gmclv1a* (*S562L*), *Gmnark* (W677*) and the *Gmclv1a Gmnark* double mutant (DM). Plants were grown at 28/25 °C or 20/17 °C. (**A)** Plant height. (**B)** Node number. (**C)** Leaf number at node 3. (**D)** Percentage of plants having at least one vein-bladed leaf; Vein-bladed phenotype were scored 4 weeks after flowering. (**E)** Pod number (including both developing and mature pods). (**F)** Nodule number per plant. (**G)** Shoot dry weight. (**H)** Root dry weight. Plant height, node number and leaf number at node 3 were measured 4 weeks after sowing; n = 9–13. Nodule number, shoot and root dry weight were measured 3 weeks after sowing; n = 6. Error bars indicate SE. Nd = ‘not detected’. Different letters above the bar represent statistically significant differences (Duncan test; *P* ≤ 0.05).

To determine whether *GmCLV1A*-controlled phenotypes were affected by temperature, wild type, *S256L* and W677* plants were grown at 28/25 °C or 20/17 °C (sub-optimal temperature) over a day-length regime of 13–15 h. At 28/25 °C, *S562L* and W677* mutant plants exhibited a similar height (Fig. 8A), but developed significantly fewer nodes (Fig. 8B), and had a significantly reduced shoot and root biomasses, compared with wild type plants (Fig. 8G and H).

At 20/17 °C, *S562L* plants were significantly taller than wild type and W677* plants (Fig. 8A). However, they produced a similar number of nodes compared with the wild type (Fig. 8B) and had a similar shoot and root biomass (Fig. 8G and H). Sub-optimal temperature delayed the flowering onset similarly in all genotypes, but caused *S562L* mutant plants to form significantly fewer pods (Fig. 8E).

Regardless of temperature, *S562L* plants formed significantly more leaves at node 3 than both wild type and W677* plants (Fig. 8C). *S562L* was also the only genotype tested to exhibit the novel vein-bladed phenotype (compare to Fig. 5C), with 70 and 80% of *S562L* mutant plants having at least one leaf displaying the phenotype at 28/25 °C and 20/17 °C, respectively (Fig. 8D). Mutant *S562L* plants also displayed more branching at the basal node compared with both wild type and W677* plants at both temperatures investigated (data not shown). *S562L* plants exhibited a normal nodulation-response to nitrogen, with no nodules detected on the well-fertilized plants at either temperature tested (Fig. 8F), whereas W677* plants exhibited the classical nitrate-tolerant supernodulation phenotype of *Gmnark* mutant plants^29, 55^. Besides temperature, day length (16 h/8 h vs. 10 h/14 h day/night) also affected the intensity of the phenotype, with short day length significantly increasing the frequency and severity of the phenotype (data not shown).

### A double mutant of *Gmclv1a* (*S562L*) and Gmnark (W677*)

To determine whether *GmCLV1A* and *GmNARK* function in the same pathway, a cross between the *Gmclv1a* and *Gmnark* mutants (W677* and *S562L*) was conducted and a verified double mutant was isolated.

In the F_2_ generation, 102 plants from a single F_1_ parent showed the predicted Mendelian ratio for two unlinked genes (9:3:3:1). The F_1_ plant was genotyped and both mutant SNPs were detected. Twenty-three F_2_ plants showed a supernodulating phenotype and were homozygous (by SNP determination and phenotyping) for *Gmnark* and 79 plants exhibited normal nodulation (χ^2^ = 0.33, *p* > 0.05). Six of the 23 supernodulating plants were homozygous for the *Gmclv1a* mutation (χ^2^ = 0.014, *p* > 0.05).

*Gmclv1a Gmnark* double mutant plants maintained the supernodulation phenotype characteristic of their *Gmnark* single-mutant parent (Fig. 8F). They developed fewer nodes, with a similar growth rate to wild type and the *Gmnark* mutant, but slower than the *Gmclv1a* mutant, at 20/17 °C (Fig. 8A and B). Double mutant plants produced similar leaf numbers as wild type on node 3 at both temperatures, but they had fewer leaves compared to the *Gmclv1a* mutant and more than the *Gmnark* mutant at 28/25 °C (Fig. 8C). All mutants investigated produced significantly fewer pods compared to wild type at 20/17 °C, whereas only the double mutant produced significantly fewer pods at 28/25 °C (Fig. 8E). The vein-bladed phenotype was only observed in double mutants grown at 20/17 °C, with more than 20% of the double mutant plants having this abnormal phenotype (Fig. 8D).

Shoot biomass at 28/25 °C of *Gmclv1a Gmnark* mutants was significantly lower compared to wild type and all single mutants, while it was not significantly different at 20/17 °C. Double mutant root biomass was similar to all single mutants and significantly lower than wild type, whereas it did not change among the lines at 20/17 °C (Fig. 8G and H). At 28/25 °C, the *Gmclv1a Gmnark* mutant did not produce any branches at any nodes. However, at 20/17 °C, they had some branches at the cotyledonary node, which was similar to the *Gmnark* mutant, but significantly lower than wild type and the *Gmclv1a* single mutant (data not shown).

## Discussion

The soybean genome is duplicated and segmented^3^, resulting in the loss, inactivation and rearrangement of homeologous gene pairs. In the case of the *GmCLV1A* and *GmNARK* homeologues, neofunctionalization appears to have taken place, as both genes segregate as Mendelian recessives, but the presence of one is not sufficient to compensate for mutation in the other^27^. Whereas *GmNARK* has a distinct regulatory role in AON, and associated inhibition of nodule formation by nitrate, *GmCLV1A* functions in shoot development with no apparent role in AON.

A *GmCLV1A* mutant, induced by EMS and screened by TILLING, showed an environmentally susceptible phenotype characterized by stem fasciation, increased stem branching, pod abnormalities, abnormal leaf development and vein-leaf development. Furthermore, expression of *Glyma06g01940* (WUSCHEL related homeobox gene) in the shoot tip of *Gmclv1a* mutant was increased^53^, which is reminiscent of the finding in Arabidopsis where the *WUS* expression domain expanded in the *clv* SAM^10^. This indicates that *GmCLV1A* of soybean is acting through a similar component as the CLV network in Arabidopsis.

Despite the large size of the *GmCLV1A* gene, and the fact that numerous mutants have been isolated in its paralogous partner, *GmNARK*, no other mutants of *GmCLV1A* are known. This may indicate that severe mutations in *GmCLV1A* are lethal and that the *S562L* mis-sense mutation identified here, though clearly disruptive to CLV1A function, is somewhat leaky, enabling plant growth and development. Consistent with this hypothesis is the location of the mutation, which is in a putative glycosylation site situated towards the end of the LRR domain, and not in the central-cleft that represents the predicted binding site for its ligand partner^36^.

The phenotype of the *Gmclv1a* mutant (*S562L*) is influenced by temperature and day length; these environmental conditions would represent stress for the soybean cv. Forrest. It is possible that the degree of plasticity is also influenced by the truncated version of *GmCLV1A* (*GmTrCLV1A*) or even *GmNARK*.

The truncated-*GmCLV1A* encoding gene (*GmTrCLV1A*) was expressed throughout the plant and is similar to that reported upstream of *MtSUNN* in *M. truncatula* ^30^ and *PvTrNARK* ^32^. Interestingly, a comparable truncated gene was not found upstream of *GmNARK, LjHAR1* or *AtCLV1*. In Arabidopsis, AtCLV1 dimerises with other proteins to perceive the AtCLV3 peptide ligand. Similarly, a model has been proposed for *L. japonicus* where LjHAR1 might form a heterodimer with LjCLV2 or KLV to perceive a CLE peptide ligand to control nodulation^42, 43^. It is possible that *GmTrCLV1* also forms complexes with other receptor proteins in signal transduction mechanisms.

*Gmclv1a* phenotypes were fully complemented in the F_1_ generation of a cross between *S562L* × Forrest. In the F_2_ generation, *Gmclv1a* homozygous mutants produced significantly more branches at juvenile nodes (Node 1 to 3) compared with both the wild-type parent and wild-type segregants. Collectively, these findings demonstrate that the *Gmclv1a* mutation is the most likely cause of the developmental abnormalities. That some phenotypes of the *S562L* mutant were stronger in cold conditions is consistent with the function of CLV1 in Arabidopsis, where the *clv1–4* mutant exhibits a stronger phenotype when grown at cold temperatures (16 °C)^24^. Furthermore, phenotypes of *S562L* mutants were intensified under short-day, which is consistent with *Atclv2* mutants, particularly in regards to shoot fasciation^26^.

The *S562L* mutation did not affect nodulation, indicating that *GmCLV1A* does not function in nodulation control like *GmNARK* ^35, 39^. Some *GmNARK* mutations, including W677* reported here, also affect lateral root formation, reminiscent of the severe root effects seen in *Ljhar1* ^31, 56^ and *Mtsunn* mutants^30^. Interestingly, both GmCLV1A and GmNARK function in the control of cell division in nascent meristems, which is reminiscent to the function of Arabidopsis CLV1 in shoot ontogeny^14, 57^. AtCLV1 is reported to be expressed in cells across the centre of the shoot meristem, in addition to floral meristems^14^ and roots^12, 58^, and has a function in regulating stem cell population of the root apical meristem^12^, further demonstrating that these receptors often function in more than one process.

The *Gmclv1a Gmnark* double mutant displayed the classical *Gmnark* supernodulation phenotype. Double mutant plants were small, with fewer nodes and a reduced pod number when grown at an optimal temperature (28/25 °C) for soybean cv. Forrest. They also had a higher number of leaves on node 3 than Forrest and W677*, but less than *S562L*. This demonstrated that *GmNARK* has a distinct function in the regulation of nodule number, which is not complemented by *GmCLV1A*, consistent with previous reports^27^. Moreover, *GmCLV1A* has a function in regulating nodal identity that is distinct from *GmNARK*. However, *GmCLV1A* does share a function with *GmNARK* in plant growth, as all double mutant plants were significantly reduced in stature compared with the wild type and single mutant parents. This suggests that *GmNARK* influences plant architecture, previously undetected in single mutants due to the presence of a functional *GmCLV1A*. This is reminiscent of *Ljhar1* mutants of *Lotus japonicus*, which lack a *GmCLV1A* orthologue, and not only hypernodulate but are also drastically reduced in size^31, 59^.

Taken together, we demonstrated that the homeologous soybean genes, *GmCLV1A* and *GmNARK*, have neodiversified and are involved in two distinct developmental pathways, yet might also act together to maintain plant growth. One controls shoot structure locally, in an environmentally-influenced fashion, while the other acts both locally and systemically to regulate nodulation and lateral root numbers. Based on the phenotypes of the *S562L* mutant, and by analogy to *CLV1* of Arabidopsis, we propose that *GmCLV1A* functions in the control of shoot meristem activity. *GmCLV1A* appears to have maintained more of the ancestral function of *CLV1*, whereas *GmNARK* has evolved, possibly when nodulation first emerged in legumes roughly 60 million years ago.

## Methods

### Mutant isolation and protein sequence analysis

A soybean mutant population was generated by chemical mutagenesis (50 mM Ethyl methane-sulfonate for 16 h) of wild type cv. Forrest and screened for mutations in *GmCLV1A* and *GmNARK* through TILLING^60^. Genomic DNA of candidate *GmCLV1A* and *GmNARK* mutant plants was isolated from leaf tissue using the QIAGEN DNeasy plant mini-kit according to manufacturer’s instructions. (QIAGEN, Hilden, Germany). A central segment of 1,594 bp of *GmCLV1A* and 1,904 bp of *GmNARK* were amplified with specific primers: *GmCLV1A* primers; 5′-AATAACTACCTTAACGGCGCA-3′ and 5′-TCCACCACTGCCAACACTACT-3′, *GmNARK* primers; 5′-TGAGATTTCCGGCGAATCCCTG-3′ and 5′-TCCACCACTGCCAACACCAAC-3′ using the expand high fidelity PCR system (Roche Applied System, Germany). PCR products were purified using the QIAquick PCR purification kit (QIAGEN, Hilden, Germany) according to the manufacturer’s protocol. The sequences were then confirmed by sequencing. A mis-sense mutation was identified in *GmCLV1A* at amino acid position 562 and a non-sense mutation was identified in *GmNARK* at amino acid 677.

Protein domains were identified through the SMART website (http://smart.embl.de;^61, 62^. Molecular modelling of external domain of *GmCLV1A* was conducted by Phyre version 0.2^63^ available at www.sbg.bio.ic.ac.uk/phyre/ and visualised by PyMOL^64^. It also was analysed with the motif scan tool available at http://myhits.isb-sib.ch (*e.g*. for glycosylation site prediction). Phylogenetic trees were constructed using Geneious 5.6^65^, with distances between proteins calculated by neighbor joining with the Geneious tree builder program.

### Phenotypic studies

Seeds of the wild-type cv. Forrest, and mutants *S562L* and W677*, were sown in 4 L, 250 mm pots filled with a grade 2 vermiculite:sand mixture (2:1) and maintained in a glasshouse under natural illumination, approximately 11/13 h standard daylight, at 28/25 °C. All plants were inoculated with *Bradyrhizobium japonicum* strain CB1809 at sowing. After germination, the plants were given 300 ml of modified Herridge’s nutrient solution every two days^66^: KNO_3_ 2 mM; KH_2_PO_4_ 0.13 mM; K_2_HPO_4_ 0.13 mM; MgSO_4_.7H_2_O 0.5 mM; KCl 0.25 mM; CaCl_2_.2H_2_O 0.25 mM; Fe-EDTA 23.5 μM; H_3_BO_3_ 11.5 μM; MnCl_2_.4H2O 2.3 μM; ZnCl_2_ 0.2 μM; CuCl_2_.2H_2_O 0.08 μM; Na_2_MoO_4_.2H_2_O 0.025 μM. All plants were grown for 15 weeks, and then scored for leaf and branch number per node, and internode length.

### Grafting studies

For grafting studies, seeds were sterilized by soaking in 70% ethanol for 1 min then rinsed five times with sterile water. They were sown in sterilized pots containing vermiculite (grade 2) and kept in a glasshouse as previously described. After emergence they received a modified Herridge’s nutrient solution, with the KNO_3_ concentration reduced to 0.5 mM. Grafting was carried out 12 d after sowing using a wedge-shaped graft. The plants were then covered with clear plastic bags as described in Delves, *et al*.^67^, Lin, *et al*.^68^. Five days after grafting, the bags were removed and the grafted plants were inoculated with *B. japonicum* strain CB1809. Four weeks after inoculation, nodule number, nodulation index (nodulated portion of root), nodule dry weight and lateral root number were determined.

### F_2_ segregation of *S562L* × Forrest

Seeds of Forrest, *S562L* and the F_2_ of a Forrest × *S562L* cross were sown in 200 mm pots filled with potting mix supplemented with Osmocote (Scotts, Baulkam Hill, Australia). The plants were inoculated with *B. japonicum* strain CB1809 at the time of planting. They were kept in the glasshouse under 28/25 °C standard Brisbane daylight in February, and watered daily. Four weeks after planting, they were scored for number of branches per node. To distinguish between the presence of a dormant bud and an actively-growing branch only buds longer than 0.5 cm were counted as a ‘growing branch’.

### Temperature studies

For temperature studies, seeds were sown in 200 mm pots filled with potting mix supplemented with Osmocote (Scotts, Baulkam Hill, Australia). The plants were inoculated with *B. japonicum* strain CB1809 at the time of planting. They were kept in the glasshouse under 28/25 °C (normal temperature) or 20/17 °C (sub-optimal temperature) for 13–15 h day length and watered daily. Three weeks after planting six plants of each line were harvested to score their nodule number, shoot and root dry weight. The remaining plants were grown for one month following which they were scored for number of leaf and branches per node, total plant length, branch length per node and number of nodes. Only branches longer than 0.5 cm were counted as a ‘growing branch’. Four weeks after flowering, all plants were screened for leaf-like structures on the underside of their leaves. The number of pods per plant was scored at full maturity, with both mature and developing pods counted.

### Pod sectioning and microscopy

Pods were fixed in 0.5% (w/v) paraformaldehyde in 100 mM sodium phosphate buffer (pH 7) for 45 min on ice and under vacuum. They were then washed three times in sodium phosphate buffer (pH 7) at room temperature. The fixed pods were embedded in 3% (w/v) agarose and sectioned to 40 µm using a Leica VT1200S vibrating microtome (Leica Microsystems, Germany). Pod sections were stained for 30 to 60 s at room temperature in 0.05% Toluidine Blue (pH 4.5) and then rinsed two times with distilled H_2_O. Sections were viewed on a Nikon Eclipse E600 compound microscope (Nikon Instruments Inc., Melville, USA).

### Stem sectioning and microscopy

Stem samples from 4 month-old plants were placed in fixative solution (formaldehyde, glacial acetic acid and 95% ethanol, 2:1:10 v/v) under vacuum, infiltrated on ice for 10 min to enhance penetration of fixative, and then kept at 4 °C for 24 h. The samples were then dehydrated for 2 h at 4 °C using 70 and 95% ethanol and at room temperature using 100% ethanol^69^. After dehydration, the samples were placed in chloroform for 5 min and then were imbedded in paraffin wax. To soften the samples, the paraffin blocks were trimmed to exposure one side of the tissue and were placed in softening solution (1% sodium lauryl sulphate and 10% glycerol) and kept for two to three days at room temperature^70^. The samples were sectioned by hand with a razor blade. Sections were stained for 30 to 60 s at room temperature in 0.05% Toluidine Blue (pH 4.5) and then rinsed two times with distilled H_2_O. Sections were viewed on a Nikon SMZ800 compound binocular microscope (Nikon Instruments Inc., USA).

### RNA extraction and cDNA synthesis

Ten different tissues including root, shoot and leaf were collected from un-inoculated 14 day-old plants. RNA extraction was performed using the TRIzol reagent (Invitrogen, Carlsbad, USA) according to the manufacturer’s instructions. DNA contamination was removed using DNaseI (Fermentase, Burlington, Canada). Approximately 1 μg of RNA was subjected to 1 unit of DNaseI at 37 °C for 40 min. The reactions were inactivated by adding 1 μl of 25 mM EDTA (Invitrogen) and incubating at 65 °C for 10 min. RNA was converted to cDNA in a 20 μl reaction mixture containing 0.5 mM deoxynucleoside triphosphates (dNTPs), 1 μl of 50 μm oligo(dT) primers, 40 units of RNaseOUT (Invitrogen), 0.5 μg of DNA-free RNA, 1x first-strand buffer (Invitrogen), 5 mM dithiothreitol (DTT) and 100 units of SuperScript III reverse transcriptase (Invitrogen) at 50 °C for 60 min. Finally, cDNA was confirmed using *GmATP synthase* (Glyma20g25920) primers with PCR.

### Quantitative real time PCR

Primers used for quantitative real-time PCR (qRT-PCR) were designed using the online primer design program Primer 3 0.4.0 (available at http://frodo.wi.mit.edu). Sequences from the soybean genome (Phytozome version 4.0; available at http://www.phytozome.net) were used to design the primers. The sequences for forward and reverse primer for each gene were 5′-TTTGGCGTGGTGCTGTTG-3′ and 5′-CCAACACTACTGCTGCATCCG-3′ for *GmCLV1A* and 5′-ACAGGCAAGGTCCCCAAC-3′ and 5′-GCATCCGTGAATGGAACAGAG-3′ for *GmTrCLV1A*. To distinguish between them, qRT-PCR primers for *GmCLV1A* were designed on the second exon of *GmCLV1A*, which is absent in *GmTrCLV1A*, while qRT-PCR-specific primers for *GmTrCLV1A* were designed from the first exon. To ensure that the primers were specific and produced only a single band, normal PCR was run using Forrest cDNA. All primer pairs were found to amplify a single product of the correct size. Sequencing of the PCR products confirmed that primers are specific to the genes.

Relative transcript abundance was detected using SYBR Green PCR Master Mix (Applied Biosystems) on an ABI 7900HT cycler (Applied Biosystems) in 384-well plate. The 384-well plates were set up using an Eppendorf epMotion 5075 Robotic system and contained no template (water) control and reverse transcription negative (RT-) controls to verify genomic DNA contamination of the samples. All reactions were carried out in duplicate for three biological replicates. The qRT-PCR conditions used were: initial denaturation of 95 °C for 10 min, then 40 cycles of 95 °C for 15 sec and 60 °C for 1 min followed by a dissociation stage of 95 °C for 2 min to assess the specificity of the PCR. Gene expression levels were normalised to that of *GmATP synthase*, which was amplified using forward primer 5′-GCGATTCTTAAGCCAGCCTTT-3′ and reverse primer 5′-ACACACCCTGGAAACTGGTGA-3′. PCR efficiency for each sample was calculated using LinRegPCR 7.5^71^.

## Electronic supplementary material

Supporting information
