# Supplementary material for: Neodiversification of homeologous CLAVATA1-like receptor kinase genes in soybean leads to distinct developmental outcomes
Source: Sci Rep. 2017 Aug 21;7:8878. doi: 10.1038/s41598-017-08252-y (PMC5566472; doi:10.1038/s41598-017-08252-y)
Supplement: Supplementary file 1 — Supporting information [file 41598_2017_8252_MOESM1_ESM.pdf]

Supplementary information

Neodiversification of homeologous *CLAVATA1*-like receptor kinase genes in soybean leads to distinct developmental outcomes

Authors: Saeid Mirzaei[a,d], Jacqueline Batley[b], Tarik El-Mellouki[c], Shiming Liu[c], Khalid Meksem[c], Brett J. Ferguson[a], and Peter M. Gresshoff[a]*

Affiliation: [a]Centre for Integrative Legume Research, School of Agriculture and Food Sciences, The University of Queensland, St Lucia, Brisbane QLD 4072; [b]Department of Plant Biology, University of Western Australia, Crawley WA 6009, Australia; [c]Department of Plant, Soil and Agricultural Systems, Southern Illinois University, Carbondale, IL 62901, USA; [d]Department of Biotechnology, Institute of Science and High Technology and Environmental Sciences, Graduate University of AdvancedTechnology, Kerman, Iran.

- Corresponding Author; Professor Peter M Gresshoff, address as above,
- Phone: +61-7-33653550, Fax: +61-7-3346-8881, e-mail: p.gresshoff@uq.edu.au

Supplementary Table S1: PCR genotyping primers

Gene/Gene ID	TILLING Primers	Gene specific PCR Primers
GmNARK<br>(Glyma.12G040000)	F: 5’-TTCTTCCGCGGTCCAATCCCTAA-3<br>R: 5’-GCAATGTAGCCGTAGGAGCCAGCA-3’	F:5’-TGAGATTTCCGGCGAATCCCTG-3’<br>R:5’-TCCACCACTGCCAACACCAAC-3’
GmCLV1A<br>(Glyma.11G114100)	F: 5’-TTCTTCCATGGTCCAATCCCTAA-3’<br>R: 5’-GCAATGTAGCCGTAGGAGCCAGCA-3’	F:5’-AATAACTACCTTAACGGCGCA-3’<br>R: 5’-TCCACCACTGCCAACACTACT-3’
